# Plant Growth-Promoting Rhizobacteria Improve Growth and Fruit Quality of Cucumber under Greenhouse Conditions

**DOI:** 10.3390/plants11121612

**Published:** 2022-06-20

**Authors:** Gerardo Zapata-Sifuentes, Luis G. Hernandez-Montiel, Jorge Saenz-Mata, Manuel Fortis-Hernandez, Eduardo Blanco-Contreras, Roberto G. Chiquito-Contreras, Pablo Preciado-Rangel

**Affiliations:** 1 Tecnológico Nacional de México, Instituto Tecnológico de Torreón, Carretera Torreón-San Pedro km 7.5, Torreón 27170, Mexico; gerardo.zapata@uaaan.edu.mx (G.Z.-S.); manuel.fh@torreon.tecnm.mx (M.F.-H.); 2 Departamento de Agroecología, Universidad Autónoma Agraria Antonio Narro, Unidad Laguna, Carretera Periférico s/n, Col. Valle Verde, Torreón 27054, Mexico; eduardo.blanco@uaaan.edu.mx; 3 Nanotechnology & Microbial Biocontrol Group, Centro de Investigaciones Biológicas del Noroeste, Av. Politécnico Nacional 195, Col. Playa Palo Santa Rita, La Paz 23090, Mexico; 4 Facultad de Ciencias Biológicas, Universidad Juárez del Estado de Durango, Av. Universidad s/n, Col. Filadelfia, Gómez Palacio 35010, México; jsaenz_mata@ujed.mx; 5 Facultad de Ciencias Agrícolas, Universidad Veracruzana, Circuito Universitario Gonzalo Aguirre Beltrán s/n, Zona Universitaria, Xalapa 91090, Mexico; rchiquito@uv.mx

**Keywords:** rhizobacteria, PGPR, nutraceutical quality

## Abstract

Cucumber fruit is rich in fiber, carbohydrates, protein, magnesium, iron, vitamin B, vitamin C, flavonoids, phenolic compounds, and antioxidants. Agrochemical-based production of cucumber has tripled yields; however, excessive synthetic fertilization has caused problems in the accumulation of salts in the soil and has increased production costs. The objective of this study was to evaluate the effect of three strains of plant growth-promoting rhizobacteria (PGPR) on cucumber fruit growth and quality under greenhouse conditions. The rhizobacteria *Pseudomonas paralactis* (KBendo6p7), *Sinorhizobium meliloti* (KBecto9p6), and *Acinetobacter radioresistens* (KBendo3p1) was adjusted to 1 × 10^8^ CFU mL^−1^. The results indicated that the inoculation with PGPR improved plant height, stem diameter, root length, secondary roots, biomass, fruit size, fruit diameter, and yield, as well as nutraceutical quality and antioxidant capacity, significantly increasing the response of plants inoculated with *A.* *radioresistens* and *S.* *meliloti* in comparison to the control. In sum, our findings showed the potential functions of the use of beneficial bacteria such as PGPR for crop production to reduce costs, decrease pollution, and achieve world food safety and security.

## 1. Introduction

Cucumber (*Cucumis sativus* L.) is a plant of the family Cucurbitaceae which is produced worldwide in open fields and protected agriculture [1]. Cucumber fruit is widely consumed for its taste and freshness around the world, in addition, its nutritional contribution and nutraceutical properties have positive impacts on health, especially in people with diabetes, hypertension, cardiovascular, and Alzheimer’s disease [2,3,4]. Moreover, it is also a high source of fiber, carbohydrates, proteins, magnesium, iron, vitamin B and C, flavonoids, phenolic compounds, and antioxidants [5,6]. In the last few years, agrochemical-based production schemes have helped to increase the yield of the cucumber crops by three, however, synthetic fertilization has caused problems with salt accumulation in soil and increased production costs [7,8,9,10].

Currently, beneficial microorganisms have been applied in the plants as a sustainable alternative for food production [11], likewise, the combination with synthetic fertilizers has led to increased plant growth and productivity [7,12]. Plant growth-promoting rhizobacteria (PGPR) are a microorganism group able to increase the shoot and root length, improve water and nutrient absorption, and improve fruit quality and productivity in plants [13]. Undoubtedly, among the promoting mechanisms the production of phytohormone (auxin and cytokinins), volatile compounds, siderophores, atmospheric nitrogen fixation processes, and solubilized phosphate are the most important [12,14].

Moreover, the common genera of PGPR reported are *Azospirillum*, *Pseudomonas*, *Bacillus*, *Azotobacter*, *Caulobacter*, *Flavobacterium*, *Enterobacter*, among others [14,15,16]. One of the most studied genera within PGPR is the *Pseudomonas* [17]. Furthermore, the *P. paralactis* has stood out in vitro for its capacity to solubilize phosphate, indoleacetic acid (IAA), and siderophores production, however, up to now, no previous study has investigated its effect in vivo [18]. *Acinetobacter radioresistens* is another PGPR that produces IAA, it can solubilize phosphate and produce siderophores, in turn, it has promoted the growth of plants such as *Ilex paraguariensis* and *Aloe vera* [19,20]. Another one is *Sinorhizobiun* *meliloti* characterized by nitrogen fixation [21], IAA production [22], phosphate solubilization [23], and the growth-promoting of the *Medicago sativa* L. and *Lactuca sativa* L. [21]. Based on the above, the role of PGPR on plants is important and necessary to incorporate into the agricultural systems production. In addition, the evaluation of their potential for fruit production and quality is just as important, therefore, the aim of the present study was to evaluate the effect of PGPR on the growth and fruit quality of cucumber fruit under greenhouse conditions.

## 2. Results

### 2.1. Morphological Parameters of Cucumber Plants Inoculated with PGPR

PGPR inoculation of cucumber significantly increased plant growth (Table 1). The height of plants inoculated with rhizobacteria; *S. meliloti*, *P. paralactis*, and *A. radioresistens* showed significant differences with respect to plants without microorganisms. Plants inoculated with *S. meliloti* and *A. radioresistens* showed an increase in stem diameter of 36 and 30%, respectively, and an increase in dry biomass of 59 and 83%, respectively. Regarding root length, *A. radioresistens* promoted an increase of 135%. Finally, *P. paralactis* increased secondary roots by 97%.

### 2.2. Fruit Length, Diameter, and Yield

Fruit length and diameter increased significantly when plants were inoculated with the three rhizobacteria and *A. radioresistens*, increasing yield by 51.9% (Table 1).

### 2.3. Fruit Quality, Total Phenolic Contents, Total Flavonoids, Antioxidant Capacity, and Vitamin C Content

**Figure 1:**
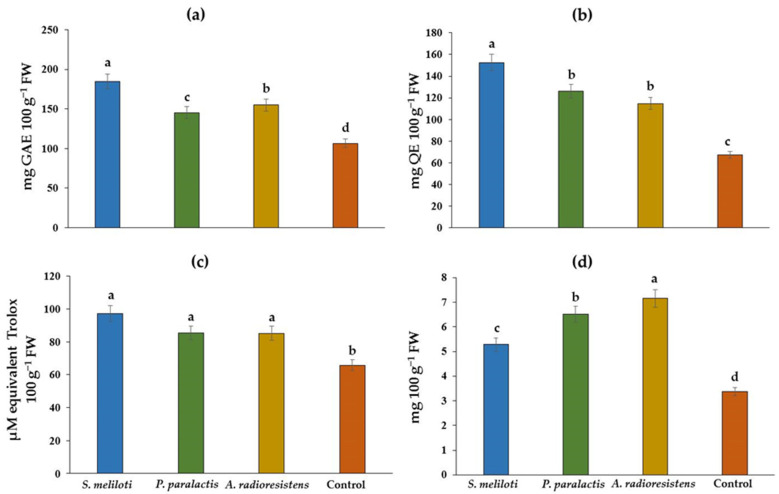
Effect of PGPR on the content of phenolic compounds (**a**), flavonoids (**b**), antioxidant capacity (**c**), and vitamin C (**d**) in *C. sativus* fruit under greenhouse conditions. Different letters indicate a significant difference (*p* < 0.05) according to Tukey’s test.

PGPR inoculation had a significant effect on the phenolic content in cucumber fruits, with the rhizobacterium *S. meliloti* promoting the greatest increase in phenolic content by 73% (Figure 1a), flavonoids by 126% (Figure 1b), and antioxidant capacity by 47% (Figure 1c). *A. radioresistens* increased the content of vitamin C by 112% (Figure 1d).

### 2.4. Total Protein

**Figure 2:**
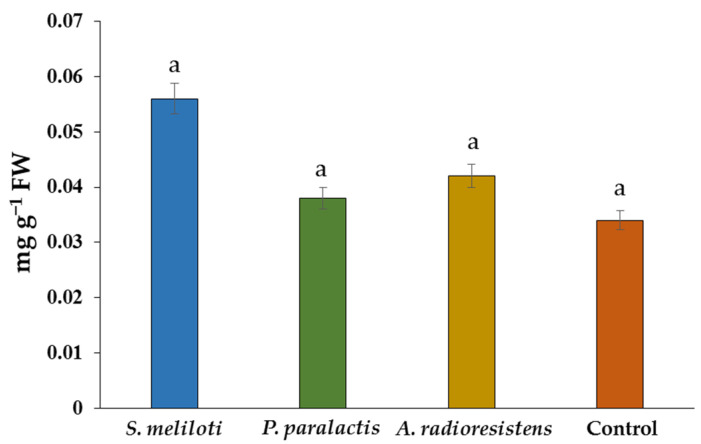
Effect of PGPR on total protein content in *C. sativus* fruit under greenhouse conditions. Data are shown as mean ± SD. Different letters indicate a significant difference (*p* < 0.05) according to Tukey’s test.

Total protein values showed no significant difference, however, inoculation with *S. meliloti* increased by 64% with respect to the control (Figure 2).

### 2.5. Rhizobacterial Population

**Figure 3:**
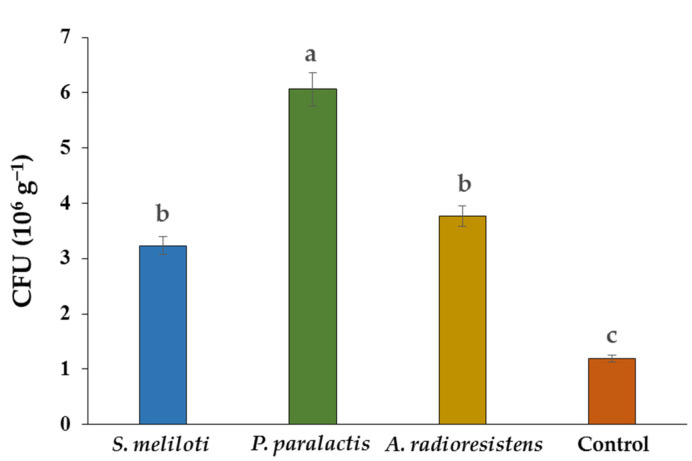
Rhizobacterial population on *C. sativus* L. Different letters indicate a significant difference (*p* < 0.05) according to Tukey’s test.

CFU counts indicated the presence of PGPR in the root of cucumber plants. There was a statistical difference between treatments, with *P. paralactis* being 409% higher than the control (Figure 3).

## 3. Discussion

*Pseudomonas paralactis*, *Sinorhizobiun meliloti*, and *Acinetobacter radioresistens*, are considered PGPR due to their capability to promote growth, development, productivity, and fruit quality [21,24,25]. The results obtained on plant height and stem diameter when using *S. meliloti* and *A. radioresistens* as inoculant in *C. sativus* plants can be attributed to the increase in root length and the number of secondary roots, implying an effect on biomass accumulation and yield. In similar studies [26], using *Bacillus velezensis* as inoculant of *C. sativus*, it was observed that the host plant modifies the root structure, which improves water and nutrient uptake, promoting a higher photosynthetic rate. It has been suggested that the modification of the root structure of a cucumber plant is related to the phytohormone indoleacetic acid (IAA) produced by *S. meliloti*, *A. radioresistens*, and *P. paralactis* [11,14,27]. In addition, it has been reported that *S. meliloti* and *A. radioresistens* possess the ability to fix nitrogen and solubilize phosphate, resulting in an increase on the number of late-stage roots and root hairs, since these elements are essential for the plant to increase photosynthetic pigments and proteins, which improves photosynthetic activity and rate, having a positive effect on plant development and a greater accumulation of biomass [28,29]. Furthermore, it has been pointed out that IAA is associated with cell division and differentiation, which improves the structure of the root system [30,31,32].

Regarding nutraceutical quality, *S. meliloti* increased the contents of phenolic compounds, flavonoids and antioxidant capacity, which can be attributed to the ability of the microorganism’s and/or its effect to induce phenolic compounds, osmolytes and organic acids in the plant, improving the availability of these compounds to be used by the plant [33]. It has been reported that in the cultivation of *Capsicum annuum*, the number of flavonoids increases when the plant is inoculated with *P. putida* because it uses quercetin and kaempferol catabolizing them as a carbon source [34], moreover, it has been observed that *S. meliloti* responds to the synthesis of proteins, mainly the Nod protein, excreting flavonoids [35]. Furthermore, if the antioxidant capacity is in dynamic equilibrium under normal growth conditions, it implies the decrease of enzymatic activity which results in the elimination of reactive oxygen, however, membrane lipid peroxidation is induced [29], and this could explain the increase of antioxidant capacity in the fruit of *C. sativus* in our results.

PGPR increases vitamin C in fruit as a non-enzymatic response to the retardation of the oxidation, also, the increase in total proteins may be due to oxidation; since the PGPR mitigate the effects of oxidation by allocating bioactive compounds to reduce the exposure of reactive oxygen species (ROS) with DNA and protein packaging, it can be inferred that the increased nutraceutical qualities of cucumber crop are due to a non-enzymatic response to natural oxidation of the plant and fruit [36].

The change in culturable bacterial subpopulation density can be attributed to the nature of each microorganism, host plant, plant genotype and environmental conditions [37], which may explain how PGPR decreases at a rate of 10^−2^ [38]. Although *P. paralactis* has been reported [39] as a PGPR due to IAA production and phosphate solubilization in vitro, this is the first study of its effect on growth promotion in cucumber plants.

## 4. Materials and Methods

### 4.1. Inoculation in Seed, Production of Seedlings, and Crop Management

The study was carried out through an agricultural cycle from Spring to Summer 2020 at Comarca Lagunera (101°40′ and 104°45′ W; 25°05′ and 26°54′ N). It was established at the Universidad Autónoma Agraria Antonio Narro, Unidad Laguna (UAAAN-UL), maintaining an average temperature of 25 °C and a relative humidity of 70%.

The genera of PGPR studied were *Pseudomonas paralactis* (KBendo6p7), *Sinorhizobium meliloti* (KBecto9p6), and *Acinetobacter radioresistens* (KBendo3p1) donated by the Microbial Ecology Laboratory of Biology Faculty from Universidad Juárez del Estado de Durango, México. After the reactivation of each bacterial strain, each bacterial strain was cultured in PBS al 0.5% [40] at 30 °C for 24 h with a constant shaking at 120 revolutions per minute (rpm). Subsequently, the concentration was adjusted to 1 × 10^8^ colony-forming units (CFU) mL^−1^ with a spectrophotometer (VELAB VE-5100UV) at a wavelength of 540 nm (Absorbance = 1.0 unit).

Cucumber seeds (Poinset 76 variety) were prepared, seeds were then disinfected with 5% sodium hypochlorite for 1 h, the polystyrene germination plates were sterilized with the same solution and washed using distilled water. In addition, the peat moss was sterilized in autoclave at 15 psi for 1 h. Prior to sowing, around 200 seeds were inoculated with immersion in 50 mL of each bacteria for 24 h [41]. One seed per cavity was deposited in germination plates which were placed in a greenhouse. After 20 days, each seedling was transplanted into 10 L black polyethylene pots containing a substrate of sand and perlite in 80:20 ratio, respectively, the substrate was previously sanitized with a 5% sodium hypochlorite solution for 72 h. Eight plants per treatment were used in a completely randomized block design.

Irrigation was based on daily evapotranspiration according to evapotranspiration requirements [42] and was applied from transplanting to 150 days after emergence from a 100% Steiner nutrient solution [43] with an electrical conductivity (EC) of 2 dSm^−1^ and a pH of 5.5.

At 55 days after transplantation (DAT), plants were re-inoculated with 15 mL of each rhizobacterium at a concentration of 1 × 10^8^ CFU mL^−1^ [40,41].

### 4.2. Parameter Evaluated

#### 4.2.1. Morphology

At 45 DAT, the plant height (cm) and stem diameter (mm) were measured and at 150 DAT, root length (cm) and secondary roots were measured. For biomass (g), three plants per treatment were taken, placed in paper bags and placed in a drying oven at 70 °C for 72 h, determining the dry biomass (g) at the end.

#### 4.2.2. Fruit Size, Fruit Diameter, and Yield

Three plants per treatment were harvested at 80 and 120 DAT to measure the fruit diameter using a digital vernier (H-7352, Uline) expressing its units in millimeters (mm), the length was measured (cm) taking the first point from the base to the appendix of the fruit and for the determination of yield the total weight of the fruit obtained by each plant (kg/plant) was recorded using a digital balance (VE-CB2000, VELAB).

### 4.3. Nutraceutical Quality, Phenolic Content, Flavonoids, Antioxidant Capacity, Vitamin C, and Total Protein

#### 4.3.1. Extracts Preparation

The extracts were obtained as follows: from three plants per treatment, two fruits were taken at random, and then crushed to obtain a compound mixture. Afterwards, 2 g of fresh pulp were placed in 10 mL of 80% ethanol in a glass tube with screw cap, leaving them in a rotary shaker (Remi RS—24BL, Jayanti Scientific, Delhi, India) for 24 h at 70 rpm at room temperature. Finally, the tubes were centrifuged at 3000 rpm for 5 min, once the supernatant was extracted; the procedure was performed in triplicate for each treatment.

#### 4.3.2. Total Phenolic Content

The total phenolic content was determined according to the Folin-Ciacalteau method [44] expressing the result in milligrams of gallic acid (GAE) per 100 g of fresh weight (FW) (mg GAE 100 g^−1^ FW).

#### 4.3.3. Flavonoids

The total flavonoids were determined by the Lamaison and library technique [45], the results were expressed in milligrams of quercetin (QE) per 100 g of FW(mg QE 100 g^−1^ FW).

#### 4.3.4. Antioxidant Capacity

The antioxidant capacity was determined with the DPPH++ method [46] and is expressed in μM equivalent Trolox 100 g^−1^ FW.

#### 4.3.5. Vitamin C Content

Vitamin C content was determined according to Hernández-Hernández [47], the units of measurement correspond to milligrams per 100 g of FW (mg 100 g^−1^ FW).

#### 4.3.6. Total Protein

Total soluble protein was determined by Bradford’s method [48] expressed in milligrams per gram of fresh weight (mg g^−1^ FW).

### 4.4. Colonies Count of Rhizobacteria

The culturable bacterial subpopulation density was carried out on a sample composed of roots per treatment (10 g), macerating the roots with a ceramic mortar to homogenize them and then gauging to 100 mL with NaCl saline solution at 0.85%. 85% of the mixture was shaken for 5 min in a Benchmark vortex and allowed to stand for 20 min, then the samples were serially diluted to 10^−4^ and an aliquot of 100 µL was inoculated in triplicate, for each treatment, in Petri dishes with 20 mL of trypticase soy agar (TSA) culture medium and 1g of copper sulfate. They were incubated at 37 °C for 48 h and colony forming units (CFU) were counted [49].

### 4.5. Statistical Analysis

The study variables were analyzed by means of an analysis of variance with the SAS 9.4 statistical package; if a significant difference was found, a comparison of means was performed using the Tukey method (*p* < 0.05).

## 5. Conclusions

Inoculation of the plants with PGPR improved height, stem diameter, root length, secondary roots, biomass, size and fruit diameter, and yield. In addition, nutraceutical quality parameters in the plants inoculated with *P. paralactis*, *S. meliloti* and *A. radioresistens* were increase compared to the control. In the future, it is necessary studies on the effects of the inoculation of bacteria consortiums on growth, productivity, and fruit quality of cucumber.

## Figures and Tables

**Table 1:** Effect of PGPR on growth of C. *sativus* cultivation under greenhouse conditions.

Treatment	Plant Height (cm)	Stem Diameter (mm)	Root Length (cm)	Secondary Roots	Dry Biomass (g)	Fruit Length (cm)	Fruit Diameter (mm)	Yield (kg/plant)
*S. meliloti*	156.27 a	4.97 a	17.21 ab	13 ab	20.07 a	21.33 a	54.44 a	5.96 b
*P. paralactis*	164.60 a	4.70 ab	16.52 ab	22 a	14.13 b	21.33 a	52.02 a	6.35 b
*A. radioresistens*	163.33 a	4.75 a	20.61 a	15 ab	23.10 a	22.67 a	56.52 a	6.94 a
Control	146.38 b	3.65 b	8.75 b	11 b	12.57 b	17.01 b	46.03 b	4.56 c

Different letters indicate a significant difference (*p* < 0.05) according to Tukey’s test.

## Data Availability

Not applicable.
